# *In Vitro* Evolution and Affinity-Maturation with Coliphage Qβ Display

**DOI:** 10.1371/journal.pone.0113069

**Published:** 2014-11-13

**Authors:** Claudia Skamel, Stephen G. Aller, Alain Bopda Waffo

**Affiliations:** 1 Campus Technologies Freiburg (CTF) GmbH, Agency for Technology Transfer at the University and University Medical Center Freiburg, Freiburg, Germany; 2 Department of Pharmacology and Toxicology and Center for Structural Biology, University of Alabama at Birmingham, Birmingham, Alabama, United States of America; 3 Department of Biological Sciences, Alabama State University, Montgomery, Alabama, United States of America; Imperial College London, United Kingdom

## Abstract

The *Escherichia coli* bacteriophage, Qβ (Coliphage Qβ), offers a favorable alternative to M13 for *in vitro* evolution of displayed peptides and proteins due to high mutagenesis rates in Qβ RNA replication that better simulate the affinity maturation processes of the immune response. We describe a benchtop *in vitro* evolution system using Qβ display of the VP1 G-H loop peptide of foot-and-mouth disease virus (FMDV). DNA encoding the G-H loop was fused to the A1 minor coat protein of Qβ resulting in a replication-competent hybrid phage that efficiently displayed the FMDV peptide. The surface-localized FMDV VP1 G-H loop cross-reacted with the anti-FMDV monoclonal antibody (mAb) SD6 and was found to decorate the corners of the Qβ icosahedral shell by electron microscopy. Evolution of Qβ-displayed peptides, starting from fully degenerate coding sequences corresponding to the immunodominant region of VP1, allowed rapid *in vitro* affinity maturation to SD6 mAb. Qβ selected under evolutionary pressure revealed a non-canonical, but essential epitope for mAb SD6 recognition consisting of an Arg-Gly tandem pair. Finally, the selected hybrid phages induced polyclonal antibodies in guinea pigs with good affinity to both FMDV and hybrid Qβ-G-H loop, validating the requirement of the tandem pair epitope. Qβ-display emerges as a novel framework for rapid *in vitro* evolution with affinity-maturation to molecular targets.

## Introduction

Following its discovery by George Smith in the early 1980's, phage display technologies have been built predominantly from DNA phage platforms, particularly that of M13 [1]–[5]. M13 is DNA-filamentous bacteriophage with a genome size of 6.4 kb [6] and have very low mutation rates that limit their use in *in vitro* evolution processes. On the contrary, RNA-based replication systems possess attractive features, including high mutation rates, high population size and short replication times, that can be exploited for rapid *in vitro* evolution [7]. Additionally, RNA-replication systems lack recombination processes that can further complicate DNA-based replication systems and technologies. Early efforts to generate recombinant RNA had limited success due to limitations in technology and RNA instability. However, with the improvement of recombinant DNA technology, and the existence of reverse transcription techniques, the generation of recombinant RNA is now straightforward. Recent advancements have led to the generation and cloning of Qβ cDNA into several stable plasmids that are able to liberate phage upon bacterial transformation [8]. The cDNA of Qβ coliphage RNA has become amenable for use in displaying random peptide libraries *in vitro* followed by *in vivo* translation and phage production.

Qβ belongs to the family of *Leviviridae* and is found throughout the world in bacteria isolates associated with sewage [9]. Of the four groups of RNA coliphages, the genome and proteins of Qβ phages have been the most extensively characterized [10]. Some representatives of these groups are: group I (f2, MS2, R17, fr) group II (GA) group III (Qβ) and group IV (SP) [11]. In this report we present a framework of peptide display and affinity maturation using Qβ phage and the integrin receptor of Foot-and-Mouth-Disease-Virus (FMDV) as a proof-of-concept for acquiring binders to a highly infectious agent with many different serotypes. FMDV, the causative agent of the most economically important infectious diseases in farm animals, has seven serotypes (O, A, C, SAT_1_, SAT_2_, SAT_3_ and Asia 1, [12]). The varied nature of the serotypes compromises the ability to control this disease using present vaccination strategies. Furthermore, the instability of currently available vaccines leaves farmers with no practical option but to slaughter, emphasizing the urgent need for new vaccines [13]. FMDV is a single stranded positive-sense RNA virus of ∼8 kilobases (kb). FMDV particles consist of four major polypeptides, three outer capsid proteins (VP1, VP2 and VP3) and a fourth smaller capsid protein (VP4). The G-H loop of VP1 is of particular interest due to its major antigenic site at the carboxyl terminal [14]–[16].

Both FMDV and Qβ have icosahedral shells of 30 nm and 25 nm in diameter, respectively [17], [18]. The Qβ genome is ∼4.2 kb surrounded by a shell of 180 coat protein molecules [17], [18]. Of these proteins, A2, A1 (known as readthrough) and the replicase are encoded by the phage genome and are important for the formation of infectious phage [19]. Due to its copy number and position [20], we hypothesize that A1 can be utilized for phage display. Phage display, previously called phage exposition, consists of an insertion of a foreign DNA fragment into the minor structural phage A1 gene to create a fusion protein, which is then incorporated into a virion that retains its infectivity and exposes the foreign peptides in an accessible form at the surface [1].

We constructed hybrid phages bearing FMDV VP1 G-H loop C-terminus that efficiently binds monoclonal antibodies directed against the antigenic loop. Furthermore, display of randomized peptides allowed *in vitro* Qβ phage selection, evolution and convergence on a displayed peptide containing a tandem amino acid sequence required for anti-FMDV monoclonal antibody recognition. The specificity, productivity, affinity and efficiency of the hybrid phage were characterized. Additionally, our data provides an insight into FMDV antigen motif representing candidates for development of vaccines for livestock.

## Materials and Methods

### Reagents

All media for bacteria culture and phages were purchased from Fisher Scientific (Pittsburgh, PA). Restriction enzymes and *T4 DNA ligase* were purchased from New England BioLabs (Ipswich, MA). Unless otherwise indicated, chemical reagents (ie. RbCl and CaCl_2_) were purchased from Sigma-Aldrich (St. Louis, MO).

### Microorganisms

*Escherichia coli* MC1016 (Invitrogen, Grand Island, NY) was used to grow and maintain plasmids. *E. coli* HB101 was used to grow and maintain pBRT7Qβ, pQβ8 plasmids and all their recombinant derivatives. Three different indicator bacteria were used for phage production and titration: K12 (*E. coli* ATCC 23725), HfrH (*E. coli* ATCC23631) and Q13 (*E. coli* ATCC 29079) purchased from ATCC. The *E. coli* bacteriophage Q-β *ATCC 23631-B1* was used as a positive wild type (wt) control in experiments.

### Antibodies

The FMDV VP1 G-H loop specific antibody, SD6, was obtained from Professor Esteban Domingo's laboratory from the Department of Virology and Microbiology of the University of Madrid, Spain. Anti-green fluorescent protein (GFP) polyclonal antibody was from Biofuture Group in Goettingen, Germany. Anti-protein tHisF and HisJ polyclonal antibodies were obtained from Professor Hans-Joachim Fritz'laboratory from the Institute for Microbiology and Genetics of the University of Goettingen, Germany.

### Hybrid phage construction

Plasmids pBRT7Qβ and pQβ8 were obtained from Professor Weber [21] and from Professor Kaesberg groups [8] respectively. These plasmids pBRT7Qβ having 7489 bp (from 1 to 7489 when restricted with *Sma*I [21]) and pQβ8 having 7393 bp (from 1 to 7393 when restricted with *Sma*I endonuclease [8]) were used for this work since they both contain the entire cDNA of the Qβ phage with different orientation. For the cloning procedure into the pBRT7Qβ plasmid, *Afl*II and *Nsi*I restriction sites were used. All the primers used to amplify foreign functional protein genes were flanked with *Afl*II or Bpu10I (forward) and *Esp*I or *Nsi*I (reverse) restriction sites (Table 1). These primers were designed to confer some important features to the foreign gene after cloning: to maintain the reading frame of the vector and to maintain the important secondary structure of phage RNA for replication transcription, translation, regulation and assembly. The *Esp*I site is absent at the end sequence of the A1 protein gene of the pBRT7Qβ plasmid. To introduce this site, DNA fragments were transiently cloned into some intermediate plasmids. After PCR, the foreign gene insert was cloned into the pCR2.1 Topo vector. The pCR2.1 vector is a linearized vector ready for direct ligation of unmodified, unpurified PCR products. This vector has a single overhanging “T” which facilitates the cloning of a *Taq* and *Phusion* amplified fragment. This vector does not contain the *Afl*II and *Esp*I restriction sites and the PCR products are cloned between two *EcoR*I restriction sites. The recombinant pCR2.1 plasmid enables amplification with higher fidelity and sequencing of the PCR fragments for further cloning.

**Table 1:** Oligonucleotides.

Name	Sequence	Functions
ABW1	atgcatttcatccttagGCTAGCttactacgacttaagatagatgaattgttcgatgttaccg	For A1 deletion with *Nhe*I and *Nsi*I restriction site reverse
ABW2	cagctgaacccagcgtatTGAacgttgctcattgccggtggtggctc	For A1 deletion forward before *Bpu*10I site used
CB191	TTACACCGCCAGTGCACGCGCGGGGATCTTGCTCACCTAACGACGAC	FMD-loop adaptor
CB192	TAAGTCGTCGTTAGGTGAGCAAGATCCCCGCGTGCACTGGCGGTG	Complementary of CB191
CB193	TTACACCGCCAGNGCANNNNNNNNNNNNNNTCACCTAACGACGA	Randomize-FMD-loop adaptor
CB194	CTGGCGGTG	Complementary of CB193 first
CB195	TAAGTCGTCGTTAGGTG	Complementary of CB193 second
CB197	ACCTTCAACCTCAATTCTTGTGTTC	For sequencing Qβ cDNA for reverse from G 2410
CB198	TGCGTGATCAGAAGTATGATATTCG	For sequencing of Qβ cDNA of end of A1 gene and insert forward from T 2083

**Figure 1:**
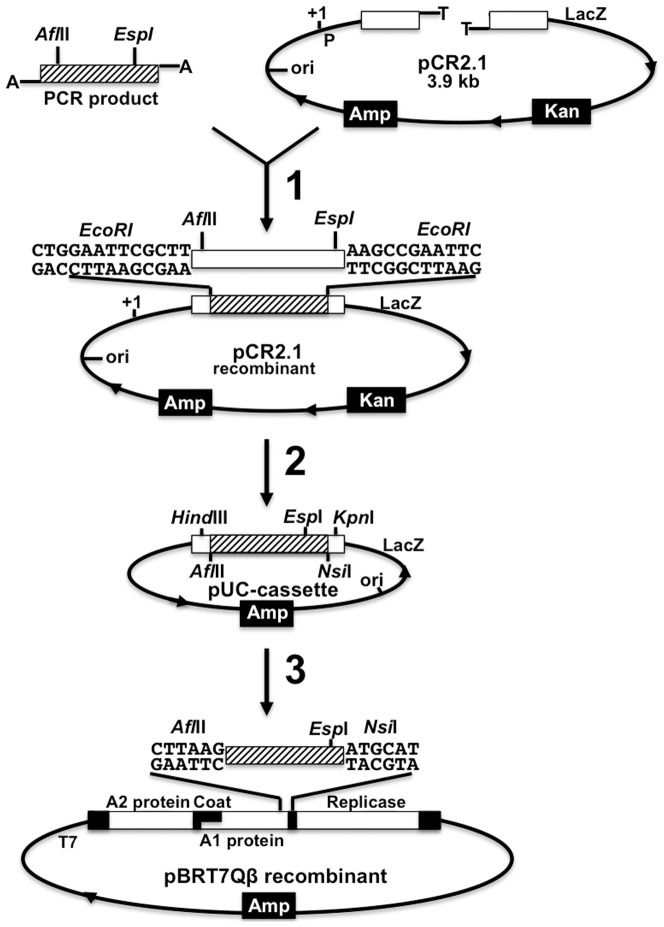
Schematic representation of the RNA phage display vector construction. General cloning procedure from PCR fragments to pBRT7Qβ with transient cloning in the pUC18-cassette working plasmid. Step 1: cloning of PCR fragment into pCR2.1 vector; Step 2: cloning of the foreign gene from PCR into the pUC-cassette (with *Nsi*I) using *Af*lII and *Esp*I sites; Step 3: Cloning of the foreign gene into pBRT7Qβ using *Afl*II and *Nsi*I. P: promoter; *Amp*: ampicillinase gene; *Kan*: kanamycin resistance gene; ori: origin of replication.

The correct insert was later cloned into a pUC-cassette vector, which allows the introduction of new restriction sites like *Esp*I. The pUC-cassette is a recombinant pUC18, containing the C-terminal of the cDNA of the A1 protein gene (from 2129–2402), which introduces the *Esp*I restriction site prior to cloning, into pBRT7Qβ. This part of the A1 gene is cloned between the *Hind*III and *Kpn*I restriction sites. Modifications aiming to add or subtract part of the foreign gene fragment to be cloned into the pBRT7Qβ plasmid were performed on the recombinant pUC18. When these manipulations were successfully performed in small size plasmids, the foreign protein gene inserts were cloned back into the pBRT7Qβ/pQβ8 plasmid as presented in Fig. 1. The recombinant pBRT7Qβ was used to generate RNA phage displaying the exogenous functional peptide. To further explore this new display technology, other functional proteins with different specific motifs that are larger than, but related to the FMDV GH-loop in the structure were also studied. These functional proteins were: the green fluorescent protein (GFP), the imidazole glycerol phosphate subunit of the synthase thermostable subunit (tHisF) and the periplasmic histidine-binding protein (HisJ).

### Deletion of A1 protein construction in pBRT7Qβ and pQβ8

To insert larger DNA fragments at the end of the A1 gene, we deleted the last 162 nucleotides of this gene keeping the interregional A1 gene and the replicase gene. These plasmids are called pBRT7QβΔA1 or pQβ8ΔA1 derived from pBRT7Qβ or pQβ8 respectively. To construct recombinant QβΔA1 plasmid, either a short portion of the cDNA of Qβ of about 420 bp was amplified with PCR or a gene part of 162 bp (between *Afl*II and *Nsi*I restriction sites) was removed and replaced by a short adaptor gene sequence. In the case of PCR, the forward primer (Table 1) used was flanked by *Bpu*10I and the reverse primer was flanked by the tag and the *Nhe*I and *Nsi*I sequences. *Nhe*I was added to monitor the cloning process. The PCR product was cloned into the pBRT7Qβ or pQβ8 plasmids using *Bpu*10I and *Nsi*I. The adaptor oligos were annealed and ligated into the *Esp*I enzyme restriction site.

### Strategy for phage production

Positive clones were transformed into *E. coli* HB 101 after sequencing using the method of Taniguchi and collaborators [22]. The supernatants of overnight clones were checked by agar overlay method for the presence of phages. The phages were amplified using an indicator bacteria cell, *E. coli* HfrH, Q13, 1101 or K12. Fresh overnight cultures (on standard nutrient agar I plate) were amplified at 37°C to reach an OD_600_ of 0.6–0.8 (after 2–3 h) and inoculated with phage suspension at a multiplicity of 3. The infected cells were incubated at 37°C by shaking (150 rpm) for 5 h. After this incubation period, the phage titer was checked and a second round of amplification was performed to scale up the phage titer according to the same procedure. At the end of amplification, indicator cells were allowed to complete the lysis by adding few drops of chloroform to the culture suspension.

### Agar overlays for spot test

This test was done according to Adam [23]. A bacteria culture was grown to log phase (OD_600_ of 0.6–0.8). A volume of 100 µl of this culture was added to 3 ml of YT-Top-agar and the mixture was poured on the surface of nutrient agar plates. The plates were left to solidify at 37°C for a few minutes. Thereafter, 4–7 µl of the phage suspension was dropped on the solidified plates. The plates were incubated at 37°C for 24 h and examined for lysis of the *E. coli* lawn where the droplet of phage suspension was placed.

### Measurement of phage yield and plaque quality

Indicator bacteria (100 µl) were infected with 100 µl of the appropriate serial dilution of phage-containing tryptone glucose yeast (TGY) solution. After 10 minutes of incubation at room temperature, 3 ml of soft-agar (TGY with 0.6% agar) was added and the mixture was poured onto plain agar plates. The plates were allowed to solidify and incubate for 16 h at 37°C. The plaque count was done following the method of Pace & Spiegelman [24]. We observed both quality and size of plaques.

### Reverse transcription (RT) PCR from plaques and/or purified phages

Phages from the clear zone of specific plaques were extracted by excising the soft media to a tube, adding 10–15 µl of H_2_O and centrifuged at 3000 rpm for 5 min to remove the media. 10 µl of the supernatant was used for RT. For the purified phage, following RNA extraction, 2–5 µg of RNA was incubated at 99.6°C with 50 pmol of reverse primers for 2 min in a total volume of 11 µl (filled with RNase-free water). This was followed by incubation on ice to allow the annealing of primers to the template. To the mixture, the following components were added: 10 µl 5×RT-buffer, 2 µl MgSO_4_ (25 mM), 1 µl 10 mM dNTP mix, 1.5 µl AMV-RT (5 U/µl) and 24.5 µl of H_2_O RNase-free.

To allow reverse transcription, the mixture was incubated at 42°C for 1 h followed by AMV denaturation at 94°C for 2 min. For the PCR reaction, 2 µl of the reverse transcriptase was used with the following protocol. The cycling protocol consisted of 25 cycles of three temperatures: 94°C, 30 s (strand denaturation), 50–57°C, 1 min (primer annealing), 68°C, 2 min (primer extension), followed by a final extension at 68°C for 7 min.

### Selection with biopanning

The antibodies were adsorbed to Xenobind™ microtiter plates (Dunn in Asbach, Germany) for biopanning. The middle wells of the plates were covered with 150 µl of the antibody solution (2.5 µg/ml in carbonate buffer: 15 mM Na_2_CO_3_ and 35 mM NaHCO_3_ pH 9.6) and incubated at room temperature for overnight. To cover the surface of the wells, a solution of 5% bovine serum albumin (BSA) in the antibody solution was added and then incubated for 1 hour at room temperature. The excess of unbound BSA was removed by washing 3 times with wash buffer (137 mM NaCl, 2.7 mM KCl, 8.3 mM Na_2_HPO_4_ 2H_2_O, 1.5 mM NaH_2_PO_4_ at pH 7.2 and 0.05% Triton X-100). A phage solution of 150 µl was then added to experimental wells of the plate and the plate was incubated at room temperature for 4 h and washed twice with wash buffer and 2 additional times with phage buffer. The experimental wells were then covered with 200 µl of *E coli* Q13 or HfrH culture (grown to OD_600_ of 0.7) and incubated at 37°C for 20 min. The bacteria culture from experimental wells was transferred into tubes as aliquots under sterile conditions after incubation. One aliquot was plated for phage titration and the rest incubated at 37°C overnight. For the next round of biopanning, 150 µl of the previous overnight bacteria and panning were used as phage solution. To further characterize phages from rounds of panning, 50 µl of phages from each round were used to extract RNA. This RNA was subjected to RT-PCR and sequencing reactions.

### Phage purification and analysis

Phage was collected using polyethylene glycol (PEG) precipitation as described in [25] with minor modifications (using PEG_8000_). Phage suspension (cell debris and phage) was incubated with 8% PEG and 0.5 M NaCl (final concentration, respectively) overnight at 4°C. The phages were pelleted by centrifugation at 3000 rpm for 20 min at 4°C (Sorvall GSA). The pellet was resuspended in phage buffer (10 mM Tris HCl pH 7.5, 1 mM MgCl2, 100 mM NaCl, 10 mg/l gelatine with 1/5 of the volume phage suspension after amplification) at 4°C for 20 min and pelleted by centrifugation at 10000 rpm for 20 min at 4°C (Sorvall RC5B, SS34). The procedure was repeated. After overnight incubation the phages were collected by centrifugation and the pellet was suspended in a small amount of phage buffer (50 µl) without gelatine. The suspension was centrifuged at 15000 rpm for 20 min at 37°C (Sorvall RC5B, SS34) and the supernatant containing phages were collected and subjected to DEAE sepharose or CsCl-gradient purification. For long-term storage the phage phase was stored in 50% glycerol at −80°C.

Another phage amplification procedure was done based on Gschwender and Hofschneider [26]. In this procedure at the log phase infected cells were incubated in high Mg^2+^ (200 mM) to inhibit cell lysis after infection. Phages were extracted from bacterial sedimentation after lysis were induced with 50 mM EDTA on ice. The suspension was adjusted to pH 9.5 by the addition of 1 M NaOH under vigorous stirring. The cellular debris was removed by low-speed centrifugation.

### Immuno-precipitation of hybrid phages against respective displayed peptide-antibody

Agarose double diffusion of Ouchterlony and Nilsson [27] was used to test the presence of foreign protein on phage surface with modifications. 1% agarose gel solution in assay buffer (50 mM Tris-HCl pH 7.5; 0.1 M NaCl) was poured into 10 cm petri dish, and allowed to solidify. Six wells were punched at equal distance from the center well. To each well 50 µl of the appropriate concentration of phages or antibody was added and incubated for 24 h at room temperature.

### Electron microscopy

A carbon-coated Formvar grid was filled with 5 µl of purified phage solution diluted to the titer of 10^9^ plaque-forming units per ml (p.f.u/ml). The solution was left on the grid for a short while and then a few drops of aqueous uranyl acetate were added. Slides were then observed under a JEOL 1200EX electron microscope.

## Results

### Tolerance of Qβ A1 gene to manipulation

**Figure 2:**
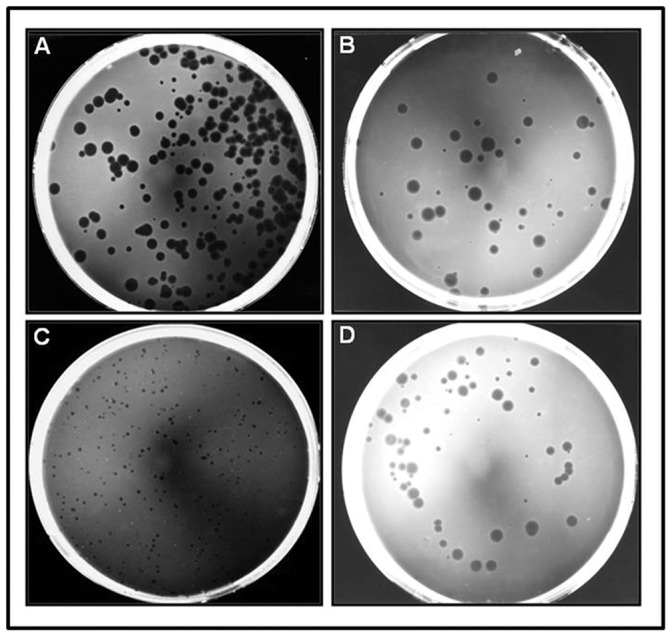
Morphology of wild type vs. hybrid Qβ phage plaques. Panel A) wild type Qβ phages; Panel B) Qβ-FMDV VP1 G-H loop phages; Panel C) Qβ-GFP rescued phages from *E. coli* SURE (expression host with F^+^) over-expressing A1-GFP protein infected with wild type Qβ. Panel D) QβΔA1 phages. All at very low multiplicity of infection (MOI), and all plates are exactly 1 day (24 hours) old when photographed.

**Figure 3:**
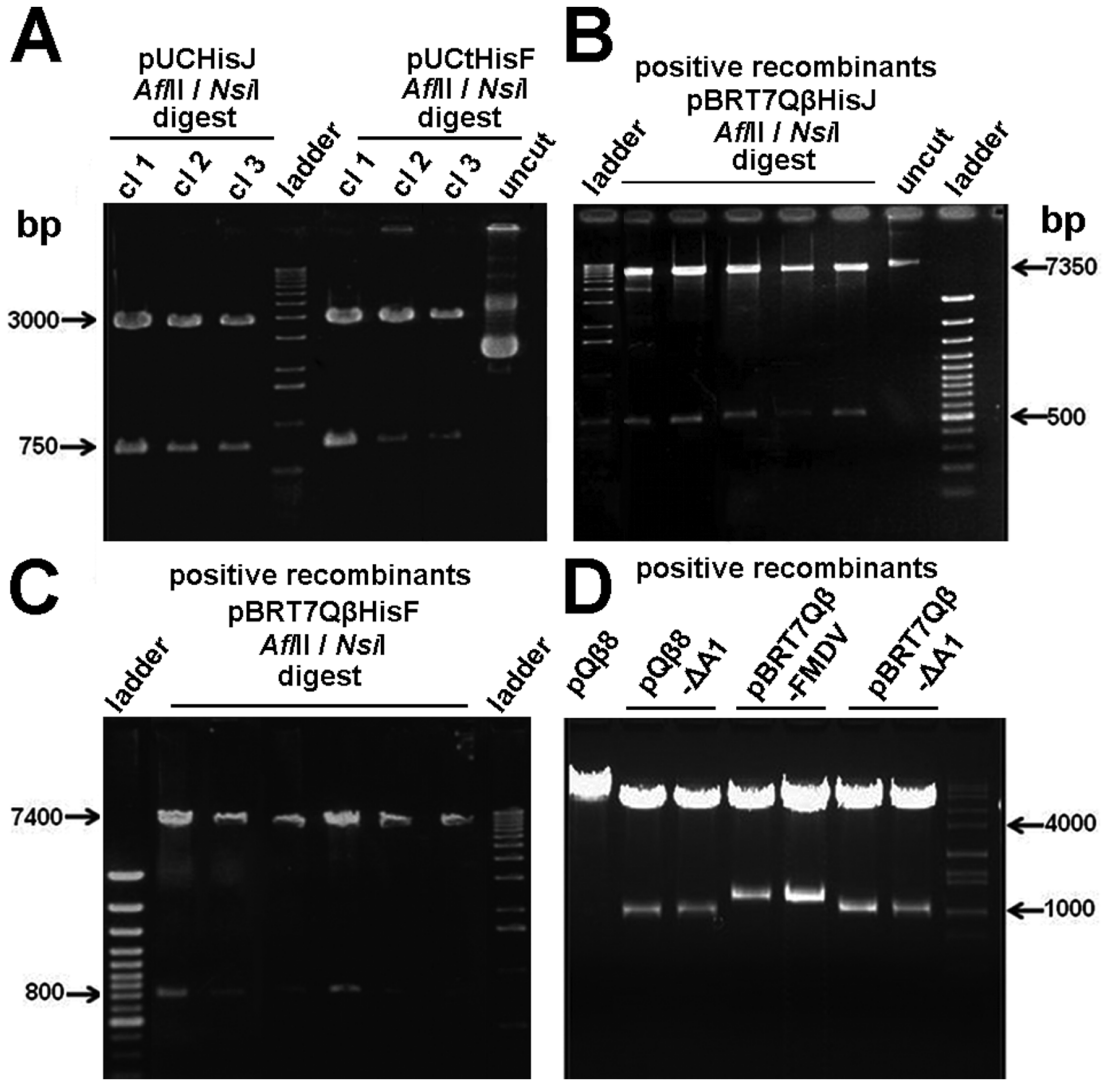
Agarose gel electrophoresis of the RNA display system vector construction. Panel A) Lanes 1–3: positive recombinant pUCHisJ plasmid clone (cl) restricted with *Afl*II and *Nsi*I; Lanes 5–7: positive pUCtHisF and Lane 8: negative clone. Panel B) Lanes 2–6: positive recombinants pBRT7QβHisJ restricted with *Afl*II and *Nsi*I; Lane 7: negative clone. Panel C) Lanes 2–7: positive recombinants pBRT7QβtHisF restricted with *Afl*II and *Nsi*I. Panel D): Lane 1: pQβ8 negative control; Lanes 2 and 3: positive recombinants pQβ8ΔA1; Lanes 4 and 5: positive recombinants pBRT7Qβ-FMDV; Lanes 6 and 7: positive recombinants pBRT7QβΔA1 all restricted with *Nhe*I. Lanes “ladder” were loaded with the 100 bp or 1 kb DNA ladder.

Initially, two variants of the pBRT7Qβ plasmid were constructed: pBRT7QβESPI and pBRT7QβNOTI. In these plasmids, additional nucleotides were added to the 3′end of A1 gene to introduce multiple cloning sites (Fig. 1). For pBRT7QβESPI, 6 nucleotides were added to introduce an *Esp*I site, and 9 nucleotides were added to pBRT7QβNOTI to introduce a *Not* I site. We tested if these extensions allowed proper DNA packing and the production of infectious-competent phage. Indeed, we show that 3 different gene fusions with A1 placed in front of the natural opal and ochre stop codons (TGA and TAA), produced phage plaques in bacterial lawns (Fig. 2). These results suggest that the 3′- end of A1 can accept minor extensions without disturbing the function of phage infectivity. We next explored the lengths of extensions and their effect on infectivity. Various DNA lengths (15–850 bp) were successfully fused with the A1 gene (Figs. 2 & 3), but only recombinant plasmids containing foreign inserted DNA with lengths between 15–300 bp produced phage plaques. These results show that the length of the inserted DNA is critically important for this novel system. Next we tested whether the 3′ end of the A1 gene is critical and important. To accomplish this, we constructed the plasmid pBRT7QβΔA1, in which non-essential sequences of the cDNA of the Qβ genome were deleted from the 3′-terminus of the A1 protein gene. Specifically, we deleted a 162 bp part of the 3′ terminus of the A1 gene (between nucleotides 2271 and 2333) and replaced it with a short adaptor gene sequence of 33 bp leaving the original intercistronic region between A1 gene and the replicase gene intact. Interestingly, these recombinant plasmids with 3′ truncations of A1, still produced phage plaques. However, further deletion of the A1 protein gene beyond nucleotides 2271 at 5′ end or 2333 at 3′ end abolished phage production. Furthermore, we tested whether the orientation of Qβ cDNA within the plasmid is critical. We created identical constructs using pBRT7Qβ and pQβ8, both of which contain the entire cDNA of phage Qβ albeit in opposite orientations. These plasmids yielded phages with similar titers to the wt, suggesting that the orientation of phage cDNA does not influence the phage production. Positive recombinant pBRT7Qβ or pQβ8 plasmids were identified via restriction enzyme (Fig. 3) prior to sequencing and transforming into Qβ for characterizing the display of foreign peptides and proteins.

### Phage production and resulting titers

**Figure 4:**
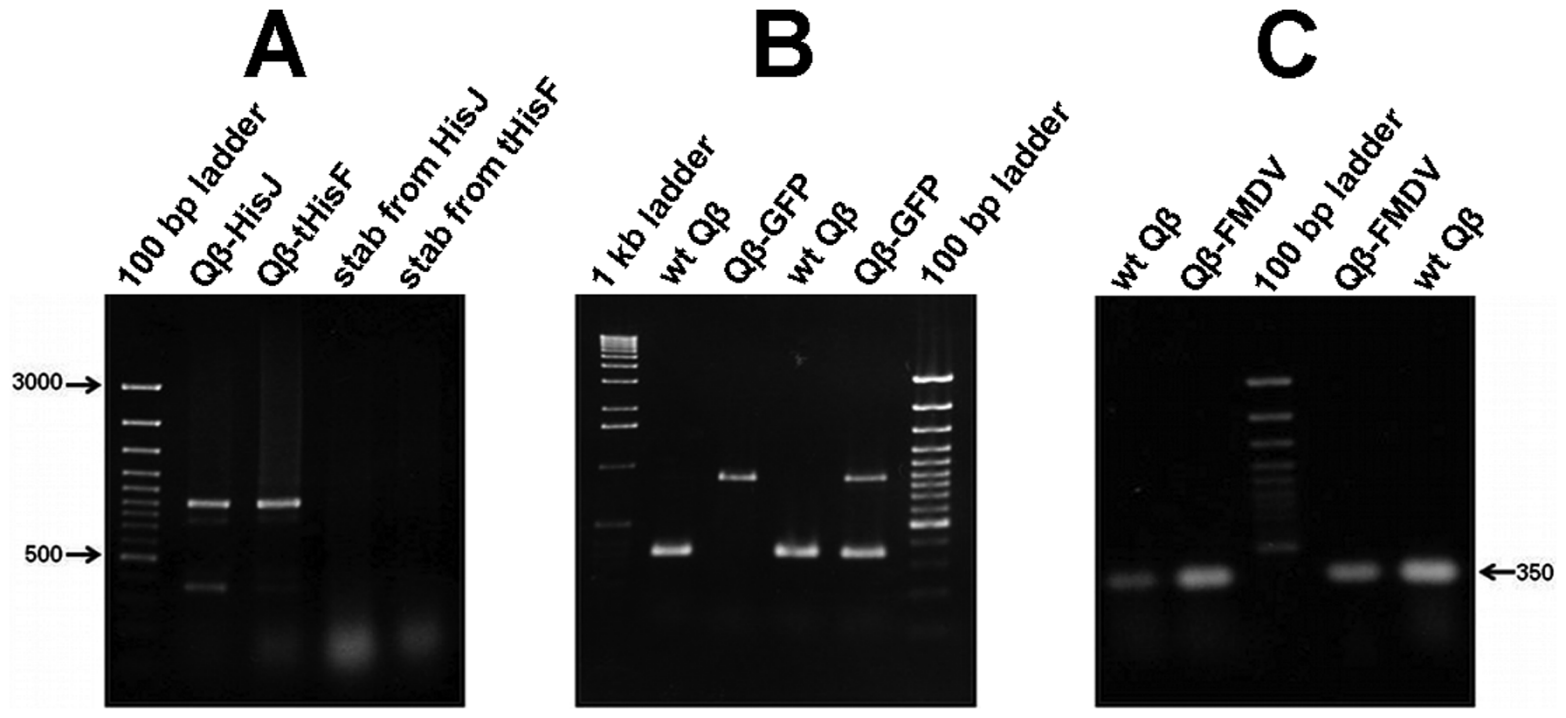
RT-PCR of RNA purified from Qβ-phage plaques. Panel A) Lane 2: Qβ-HisJ; lane 3: Qβ-tHisF; lane 4: soft agar stab from HisJ plate; lane 4: soft agar stab from tHisF. Panel B) Lanes 2 and 4: wild type Qβ; Lanes 3 and 5: Qβ-GFP. Panel C) Lanes 2 and 4: Qβ-FMDV; Lanes 1 and 5: wild type Qβ (positive control). The 100 bp and 1 kb DNA ladder were used.

To produce wild type and recombinant phages, all plasmid vectors and variant constructs were transformed into *E. coli* HB101. *E. coli* HB101 bacteria were selected because they lack the pili appendage (F^−^) necessary for Qβ absorption and infection. This insures exclusive usage of high-fidelity DNA polymerase-mediated replication of Qβ genes and prevents premature evolutionary events. Similar phage titers for both wt and recombinant phages (∼10^8^ to 10^9^ p.f.u/ml) were obtained with plaque sizes ranging between 1 mm and 3 mm in diameter in both wt and variants. The plate of phage harboring the 3′-truncation of A1 minor coat protein gene was dominated by smaller (1 mm and 70%) than larger (3 mm and 30%) sized plaques as shown in Fig. 2. However, some minor differences in plaque size were also observed. We interpret these differences as due to either the nature of the quasispecies within the phage population and/or effects associated with the insert size (Fig. 2). Next, we analyzed the phages with different sized plaques using RT-PCR and wt Qβ was used as a standard (Fig. 4). Finally, all cDNAs were sequenced to confirm the presence of the appropriate foreign gene within the hybrid phage. Results show that sequences encoding foot-and-mouth disease virus (FMDV), HisJ and HisF, appended onto Qβ-A1 allow assembly of plaque-forming phage particles containing the gene fusions.

### Efficient Qβ-FMDV phage library display and biopanning

**Figure 5:**
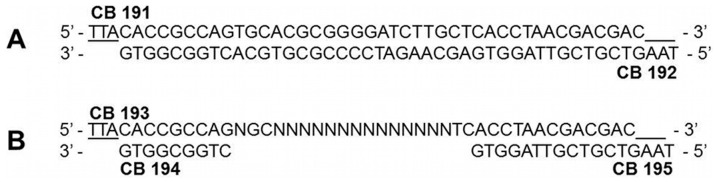
Design and schematic of FMDV VP1 G-H loop (serotype C-S8cl) oligonucleotide sequences. Panel A) Original sequence used for cloning (with *Esp*I site at both ends) into Qβ and production of phages Qβ-FMDV for guinea pig immunization. Panel B) Randomized sequence synthesis (with ends similar to Panel A) for library generation and phage population production and selection against mAb SD6.

**Figure 6:**
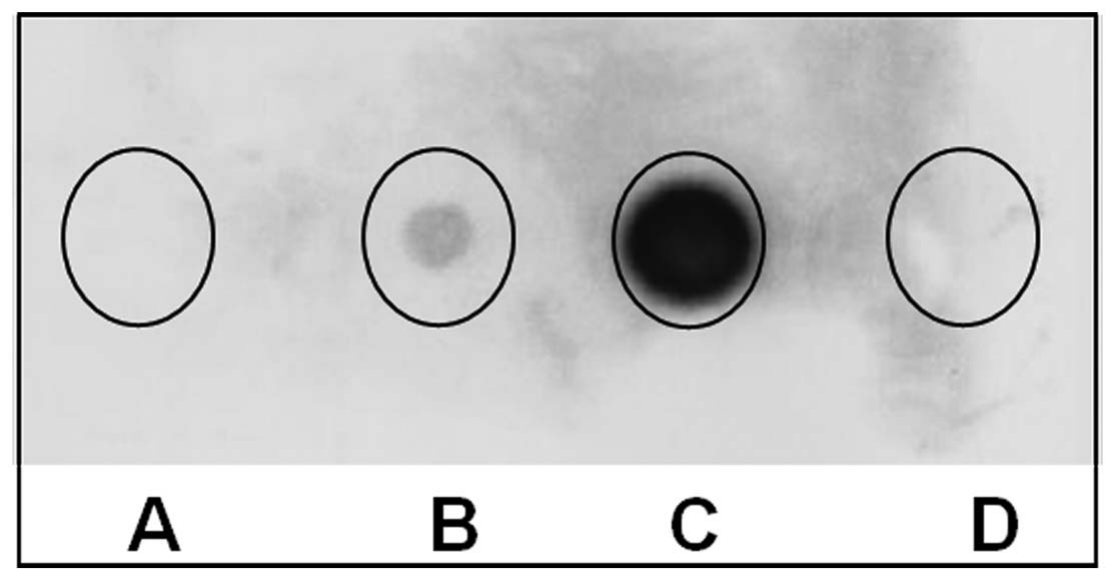
Dot blotting analysis of the FMDV VP1 G-H loop displayed on Qβ phages with SD6 mAb. Spot A: Supernatant of the Qβ wild-type culture infection (negative control); Spot B: Qβ-G-H loop phages from the supernatant of culture after 5 h of infection; Spot C: Qβ-G-H loop phages from the supernatant after overnight infection (higher concentration); Spot D: the phage buffer only as a negative control. Phages from spots A, B and C were purified by PEG_8000_/NaCl precipitation and CsCl gradient. The same pattern was obtained with the spotted corresponding crud lysat.

Rapid fitness gains are the main goal of molecular evolution and are directly proportional to the population size and the selection pressure. To mimic this process *in vitro*, we synthesized a randomized VP1 G-H loop library (YTAXA**XXX**XXHLTT) that corresponded to the immunodominant region of VP1 including the canonical RGD epitope (YTASA**RGD**LAHLTT) using three oligonucleotides CB193, CB194 and CB195 as depicted in (Fig. 5). We then cloned the randomized library into Qβ plasmids (pBRT7Qβ and derivatives) and used monoclonal antibody (mAb) SD6 as the constant selective target in a biopanning assay. Additionally, the original sequence of the VP1 G-H loop of FMDV serotype C clone C-S8cl was cloned into pBRT7Qβ using the annealed oligonucleotides: CB191 and CB192 (Fig. 5) as a control. Recombinant plasmid (pBRT7Qβ-FMDV), derived from the previous vector harboring the VP1 G-H loop fused with A1 within the phage cDNA was used to transform *E. coli* HB101. Only 50–55% of these clones produced phage plaques (not shown). Positive VP1 G-H loop clones were validated through RT-PCR and sequencing. We further confirmed the presence of the G-H loop of the hybrid phages through dot blot using mAb SD6 (Fig. 6). We further randomized the G-H loop to form a library, which was directly ligated with A1 of cDNA of Qβ. The Qβ-FMDV phages were found to produce clear plaques in all wt Qβ natural hosts namely: *E. coli* K12, Q13, and HfrH. As before, we recapitulated our data in HB101, showing that, as with wt phage, Qβ-FMDV phage can be propagated in the other *E. coli* strains. There were no significant differences in the yield of phage particles between Qβ-FMDV and Qβ. Finally, these Qβ-FMDV phages were amplified in Q13 cells (chosen among other Qβ hosts) and a high titer (10^9^ p.f.u./ml) was obtained and purified by ultracentrifugation on CsCl gradient for guinea pig immunization.

### Non-canonical FMDV epitope

**Figure 7:**
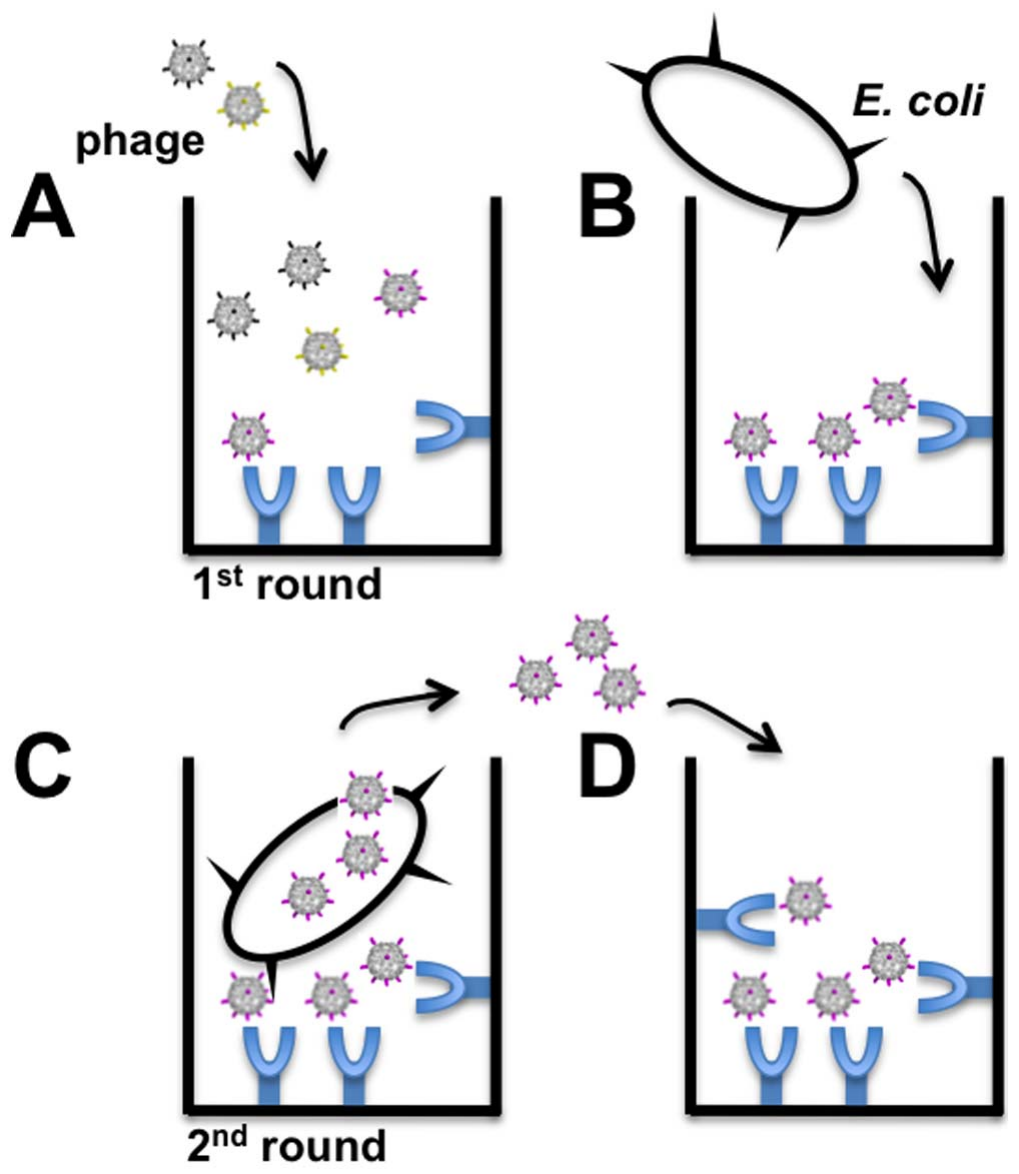
Schematic of biopanning assay with Qβ phage derivatives without the usual acidic elution. A) A population of phages displaying the library of interest (here randomized VP1 G-H loop) was added to the well of a plate pre-coated with the desired target (in this case, mAb SD6 covalently immobilized with F_c_ region). B) Indicator *E coli* are added to the well after phages having low-affinity to the target are removed. C) High-affinity phage bound to target can infect *E. coli* Q13 by adsorbing and injecting its RNA via the F^+^ pilus. D) Phages newly obtained after indicator *E. coli* infection were transferred to new wells containing the immobilized target for the next round of biopanning.

**Figure 8:**
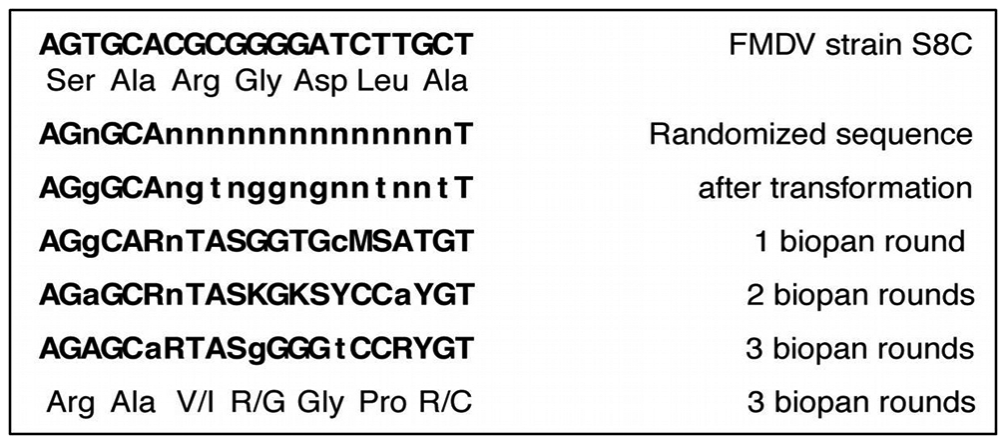
Sequences comparison of the randomized FMDV G-H loop displayed after three rounds of Biopanning. The first line up is the original loop motif ***Arg-Gly-Asp*** of VP1 G-H loop of FMDV strain S8C. On the left hand side under the original sequence are the sequences obtained after a round of biopanning. The low case letters show the different between the sequences. The last line is the expression of the evolution of the original motif sequence on the 3^rd^ round shown the maintanance of ***Arg-Gly*** and change of the last amino acid.

To gain fitness, the synthesized library of the G-H loop was selected using a modified biopanning protocol [1], [2], [28] with mAb SD6. This modified protocol selects and amplifies phage while avoiding acidic elution of phages selected (Fig. 7). We reasoned that removal of an acidic elution step would enhance phage viability and the overall efficiency of *in vitro* evolution. Additionally, media containing the indicator bacteria *E. coli* Q13 grown to the log phase was added directly to the plate to further enhance survival of hybrid phages. After each round, an aliquot of phage was used for RT-PCR and the resulting DNA sequence was compared to the wild type sequence (Fig. 8). The sequence comparison of the randomized VP1 G-H loop after six rounds of biopanning revealed a shift of mAb SD6 binding motif from *Arg-Gly-Asp* to *Xxx-Arg-Gly*. Due to the preservation of the *Arg* and *Gly* in all three rounds of biopanning, we conclude that this tandem pair is essential for mAb SD6 binding. The third amino acid was substituted without disturbing the binding capacity of the peptide to the mAb SD6. Over 80% of glycine exposed by the phage was in contact with the mAb SD6. This clearly shows that arginine and glycine were not just only together in the antibody binding motif but were representing optimized amino acid from a randomized pool.

### Immunization characterization of Qβ-FMDV phages

**Figure 9:**
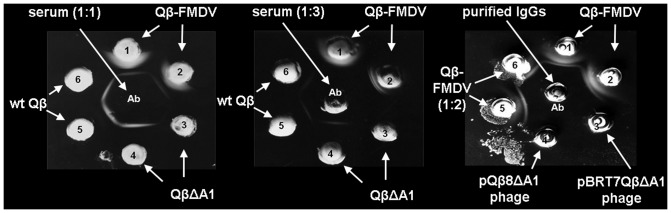
Ouchterlony double diffusion assay. A) Wells 1 and 2 represent Qβ-FMDV phages; wells 3 and 4 represent QβΔA1 phages; wells 5 and 6 represent wild-type Qβ; center well contains polyclonal serum from immunized guinea pig (labeled “Ab”). B) Same as panel A but with 1/3 of the serum concentration. C) Wells 1 and 2 Qβ-FMDV are the same as panel A; wells 5 and 6 contain half the phage titer of wells 1 and 2; well 3 represents phages from pBRT7QβΔA1 and well 4 represents phages from pQβ8ΔA1; center well contains IgGs purified from serum panel A and B (labeled “Ab”). The line of precipitation is visible as a white haze forming a half-circle around some of the wells in the experiments.

To assess the immunogenicity of the hybrid phages, guinea pigs were immunized with purified Qβ-FMDV phages. The serum obtained after immunization tested positive for FMDV antibody (Professor Esteban Domingo, personal communication). We validated this finding with a qualitative Ouchterlony assay (27) using serum obtained from immunization with Qβ-FMDV phages against the same phages on one hand, then Qβ wt. In this assay, antibody and antigen solution are placed in nearby wells cut out of a thin layer of agarose and allowed to diffuse toward each other forming a visible line of precipitation where they meet. The lines produced by the two adjacent wells containing Qβ-FMDV and Qβ wt join together in a pattern of partial identity (Fig. 9A). More specifically, at least two epitopes on Qβ-FMDV were recognized by the serum antibody. A similar result was obtained with immunoglobulin purified from the serum with protein A affinity column (results not shown). The fractionated Igs from the column did not react with phage displaying the C-terminal deletion of A1 (pQβ8ΔA1; Fig. 9C). Furthermore, the thickness of the precipitation line in the double diffusion was reduced with the reduction in the serum amount shifting towards the phages wells (Fig. 9B). The second line of precipitation close to the phage wells was reduced with half of the phages titer (Fig. 9C). The additional line of precipitation on Ouchterlony assay plate showed the presence of an epitope on Qβ-FMDV that is absent on both wt Qβ and pQβ8ΔA1. We conclude that this additional line of precipitation can only be the VP1 G-H loop exposed on the exterior surface of Qβ. This result illustrated and clarified the heterogeneity and specificity of the serum obtained from immunized guinea pigs.

**Figure 10:**
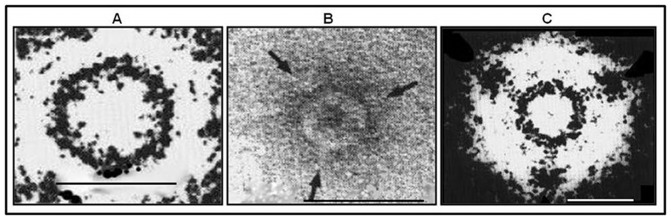
Field light micrograph of modified Qβ. A) Unlabelled Qβ virion: the original particle projection obtained with conventional transmission electron microscopy of a negatively stained sample was treated by Marham rotation 3 times 120\grad\intervals and printed at reversed contrast; Magnification Bar: 50 nm. B) Negatively stained Qβ, modified at A1 gene products by additional of FMDV G-H loop motif decorating the corners by IgG of mAb SD6 against VP1 G-H loop motif at 120\grad\intervals (arrows); Magnification Bar 25 nm. C) Phage particles projection depicted in B was treated by Makham rotation and printed at reversed contrast; arrows showing antibody against integrin motif attached to the corners of the virus particle; Magnification 25 nm.

Finally, Qβ-FMDV presence and display was validated using negative stain electron microscopy that shows the presence of antigen-antibody interaction. The wt phage particle was found with a clear zone around its particles (Fig. 10A), while Qβ-FMDV phages displaying the G-H loop of FMDV showed dots decorating its surface by mAb SD6 (Fig. 10C). We theorize that, these dots represent the position of A1 fused with the G-H loop of FMDV on the exterior surface of phage Qβ (arrow). The combined results of animal immunization, EM and serological assays, suggested that, on the exterior surface of Qβ phages, there can be exposed epitopes which may be used to induce the production of specific antibody.

## Discussion

Current phage display technologies have been exclusively designed using DNA-based phage platforms such as M13 [1]–[5], [28]. Use of such systems for library screening purposes requires highly diverse starting libraries encoding the displayed proteins since *in vitro* evolution is difficult, due to the relatively high-fidelity of proof-reading bacterial DNA polymerases. The work of Drake [29] showed a surprisingly consistent overall genomic mutation rate for DNA-based replication in plants, yeast, bacteria and bacteriophage. When the mutation rate per base was multiplied by the genome size, the mutation rate per genome for these DNA-based replicating organisms fell within an astonishingly narrow range (0.0022–0.0046; Table 2). Genomic replication error rates for RNA-based replicating MNV11RNA and bacteriophage Qβ were 7× and ∼500× greater, respectively, revealing a high degree of inherent genomic evolution potential. Compared to M13-based mutation rates, Qβ has a 420× greater mutation rate per base, indicating a major advantage in utilization of Qβ for *in vitro* evolution. Moreover, relatively harsh acidic elution procedures common to DNA phage systems add another obstacle in developing a practical system for *in vitro* evolution. The RNA phage Qβ possesses key features that can be exploited for *in vitro* evolutionary display that overcomes both obstacles [30]. We describe here a framework for Qβ display that allows a robust *in vitro* evolution at the lab benchtop.

**Table 2:** Table adapted from Drake [29].

Species name	Genome size (bases)	Target	Mutation rate (per base)^a^	Mutation rate (per genome)
*N. crassa*	4.19×10^7^	*ad-3AB, mtr*	7.2×10^−11^	0.0030
*S. cerevisiae*	1.38×10^7^	*URA3, CAN1*	2.2×10^−10^	0.0031
*E. coli*	4.70×10^6^	*lacI, hisGDCBHAFE*	4.6×10^−10^	0.0022
bacteriophage M13	6.41×10^3^	*lacZ*α	7.2×10^−7^	0.0046
bacteriophage *λ*	4.85×10^4^	*cI*	7.7×10^−8^	0.0038
bacteriophage T2	1.60×10^5^	*rII*	2.7×10^−8^	0.0043
bacteriophage T4	1.66×10^5^	*rII*	2.0×10^−8^	0.0033
MNV11RNA^b^	86	itself	3×10^−4^	0.026
bacteriophage Qβ^b^	4.2×10^3^	replicase	3×10^−4^	1.9
Taq polymerase	n/a	n/a	2×10^−5^	n/a

a In the cases where multiple targets were measured, the average is presented.

b Taken from Domingo [48].

Developing Qβ phage for peptide display included four steps. Firstly, the DNA sequence coding for the displayed peptides or proteins was fused to the end of the A1 minor coat protein gene in the DNA plasmids utilized. A1 is essential for infection, but specific roles in the Qβ virion cycle have not yet been elucidated. Our results indicated that the C-terminus of A1 (nucleotides 2271 to 2333), plays only a minor role in function and is consistent with a previous report describing the importance of the N-terminus [31]. Secondly, recombinant plasmids were sequenced and transformed in bacteria cells to produce a population of phages. Thirdly, hybrid phages were subjected to several rounds of selection and amplification to optimize interactions with the target. Fourthly, selected phages were analyzed for the presence of the correct recombinant RNA and the ability to display the appropriate protein. We examined the limits of the sizes of displayed proteins by attempting display of HisJ (726 bp), HisF (753 bp) and GFP (714 bp). Unfortunately, all three proved too large for Qβ, as evidenced by poor infectivity and/or plaque formation, even when fused to full-length A1 or A1 3′ truncation. The transformation efficiency of these constructs dropped with the increasing size of the insert but could be somewhat improved by using the rubidium chloride method with heat shock at 43.5°C, in contrast to conventional methods [32]. The Qβ phage remains functional (able to absorb and infect) with up to 60 nucleotides inserted into the A1 gene. The phage can also function with 162 nucleotides removed from the 3′ end of the A1 gene. These observations together give the A1 gene a total loading capacity of ∼222 nucleotides (74 amino acids protein). When more than 300 nucleotides of foreign gene were inserted, no viable, stable, infective replicable phages were obtained. Although spontaneous Qβ particles have been previously obtained with the A1 protein extended to 195 amino acids [33], neither replication competency nor recombinant RNA-genomic packaging limits were determined. An enormous drop in phage viability was found using MS2 phage to display five amino acids (Ala-Ser-Ile-Ser-Ile) on the exterior surface [34] revealing potential limitations on the secondary structure of the recombinant RNA on its influence on the replication, regulation and assembly to form viable phage [35].

Successful transformation and subsequent amplification was achieved with the A1 3′ truncation plasmid and the 14 amino acid G-H loop of FMDV plasmid. FMDV is a particular threat for animal livestock worldwide and the large number of serotypes and diversity of strains make the development of a universal vaccine very challenging. Through its RNA replication system, FMDV has a very high mutation rate which allows the virus to escape drug suppression [7]. Considering the highly mutable character of RNA viruses, we reasoned that a vaccine system that can also exploit this feature of RNA viruses would be highly valuable. The displayed G-H loop was found to occupy the corners of the icosahedron of Qβ as visualized by negative-stain electron microscopy, which corresponds to its natural positions in the FMDV structure [36]. The recombinant A1-G-H loop was found to decorate the phage at 12 copies per virion, indicating that the wt A1 protein must also have 12 copies per Qβ phage. Prior to these results, the exact copy number of the A1 protein was not well known but was estimated to be between 3–7% of total phage protein [20], [37], [38]. An octa-peptide of β-tubulin motif was also found to decorate the 12 corners of the Qβ icosahedron (unpublished data). This is in contrast to the commonly used phage M13 where any fusion to the minor coat protein gene, pIII, would display the foreign peptide on only one side of the phage [39].

We next exploited the high error-rate of the RNA-dependent RNA polymerase of Qβ to test the feasibility of using this system for *in vitro* evolution. Transforming a completely degenerate DNA that appended seven randomly encoded amino acids to the A1 protein, with a total theoretical diversity of 2×10^14^, has resulted in a six amino acid peptide library with an actual diversity of 2×10^8^. In only six rounds of biopanning and selective pressure in the presence of immobilized SD6 antibody, we isolated the Qβ phage that contained a conserved tandem pair, *(Arg-Gly)*, that is essential for mAb SD6 recognition. The main motif of the G-H loop was known to be Arg-Gly-Asp that recognizes and binds to mAb SD6 [14], [15]. Upon randomization and selection, the Arg-Gly motif was found to be enough for binding to the same mAb SD6, amongst the spectrum of variants generated. Panning with Qβ-FMDV phages has a double advantage of binding to antibody (selection) and being amplified without conventional acidic elution of the phage. The phages were selected by the A1 protein extension which does not hinder adsorption via the pilus, allowing the phage to inject RNA. To mimic the natural infection of Qβ during panning, prevents acidic elution and neutralization steps previously needed before further enrichment and amplification [40]–[42]. This newly developed panning method can be exploited in new antibody selection from a pool of mRNA since it is very similar to the natural one without intervention of rough chemicals like in the case of most DNA phage display technologies.

Serum from guinea pigs immunized with Qβ-FMDV exhibited cross reactivity with intact FMDV as well as the hybrid Qβ phage displaying the G-H loop in a qualitative Ouchterlony assay. The fact that Qβ exist as quasi species with very high mutation rate [43]–[48] is a double advantage for Qβ-FMDV hybrid phages which contain a pool of antigens (quasispecies with mutant spectra) important for immunization and potential vaccine. A pool of different antigen FMDV G-H loop strains can be exposed on Qβ surface and used as vaccine.

In conclusion, we have developed a peptide library display system using the Qβ RNA-coliphage that efficiently mimics evolutionary adaptation and affinity maturation. A randomized G-H loop of the FMDV VP1 protein was exposed on the exterior surface of Qβ and selected against the G-H loop mAb SD6. Guinea pig serum immunized with the hybrid phages (Qβ-FMDV) contained immunoglobulin specific to FMDV and the Qβ-FMDV hybrid phages. These hybrid phages could principally serve as good candidates for FMDV vaccine development. Robust viability and infectivity was achieved with a C-terminal A1 deletion that maintained 12 copies per virion. Current size limitations of display are ∼74 amino acid peptide/protein domain since larger domains (e.g. GFP) could not be displayed. With further optimization of A1-appended sequences, Qβ display of larger protein domains may eventually prove possible. The Qβ *in vitro* evolution platform we describe here may be readily adapted for the development of nanotechnology including novel biosensors, therapeutics, immunization reagents or crystallization scaffolds.
