# Correction: *In Vitro* Evolution and Affinity-Maturation with Coliphage Qβ Display

**DOI:** 10.1371/journal.pone.0199953

**Published:** 2018-06-26

**Authors:** Claudia Skamel, Stephen G. Aller, Alain Bopda Waffo

The following information is missing from the Funding section: The authors acknowledge the funding received from the American Cancer Society Institutional Research Grant (IRG-60-001-53).
